# When Biobanks Meet Religion: Association Between Religiosity and Attitudes of Polish Medical Students Toward Biobanking of Human Biological Material for Research Purposes

**DOI:** 10.1007/s10943-023-01932-2

**Published:** 2023-10-17

**Authors:** Jan Domaradzki, Dariusz Walkowiak

**Affiliations:** 1 Department of Social Sciences and Humanities, Poznań University of Medical Sciences, Rokietnicka 7, St., 60-806 Poznan, Poland; 2 Department of Organization and Management in Health Care, Poznań University of Medical Sciences, Poznan, Poland

**Keywords:** Attitudes, Biobanks, Biobanking, Biobank research, Future healthcare professionals, Human biological material, Medical students, Religion, Tissue donation

## Abstract

While biobanking is expanding globally, the empirical evidence concerning the impact of religion on future healthcare professionals’ awareness and willingness to donate biospecimens for biobank research is lacking. To understand how medical students’ religious beliefs can fuel their questions regarding how biospecimens would be stored, cared for, and used, we conducted a survey among 1500 medical students at Poznań University of Medical Sciences. Our findings suggest that, while both religious and nonreligious students supported the idea of biobanking of human biological material and were willing to donate for research purposes, nonreligious students felt more positive toward biobanking, supported the idea of establishing biobanks in Poland more often, and were more eager to donate most types of tissues and to participate in biobank research. Religious beliefs were also associated with medical students’ perception of benefits and risks resulting from biobanking, perceived trust toward various biobank institutions, and preferred type of consent.

## Introduction

Although the idea of biobanking is not new, since (human) biological samples have been collected and stored in many countries for over 100 years, genetic engineering and the sequencing of the human genome has revolutionized the use of biospecimens for research purposes; it has opened the door to new methods of disease study and encouraged a new era of biomedical research (Coppola et al., 2019; De Souza & Greenspan, 2013; Hartman et al., 2019; Yaghoobi & Hosseini, 2021). Consequently, over the past few decades, both the public and private sectors around the world have begun investing in large-scale programs for establishing new biobanks, which have become an important tool for personalized medicine and scientific progress (Kinkorová, 2016; Olson et al., 2014).

However, since clinical research conducted in biobanks involves not only the collection of human specimens (e.g., DNA, blood, cells, tissue) but also related personal and health information (health records, family history, lifestyle, genetic information), biobanking differs significantly from traditional tissue collection and clinical trials. Consequently, biobanks generate many ethical, legal, and social concerns, including data protection, informed consent or profit-making, and benefit-sharing, which can discourage people from donating their biosamples for research purposes (Bledsoe, 2017; Domaradzki, 2019; Haga & Beskow, 2008; Hoeyer, 2012).

Simultaneously, although socioecological context has been recognized as an important component that can influence the public’s attitudes toward biobanking, previous research focused on the influence of various demographic, socioeconomic, and psychosocial factors, such as age, gender, race/ethnicity, education, or personality traits (Domaradzki & Pawlikowski, 2019; Pawlikowski et al., 2022; Sedlár & Grežo, 2022). Meanwhile, one key factor that can affect biobanks is religion (Eisenhauer & Arslanian-Engoren, 2016; Toh et al., 2021; Yeary et al., 2020). Particularly in countries with a strong regard for religion, religious beliefs surrounding the human body and its parts can affect one’s perception of body organs, donation, and scientific research, and can influence donors’ decisions regarding biobanking (Ahram et al., 2014; De Vries et al., 2016a; Goddard et al., 2009; Hasrizul et al., 2017; Igbe & Adebamowo, 2012; Merdad et al., 2017).

However, religion can serve as a double-edged sword: while it can facilitate advances in biobank research and recruitment of potential donors, it can also undermine this process. Indeed, since most religions emphasize values of altruism, charity, acting for the greater good, and helping others, these beliefs can lend support for organ donation and participation in biobank research, which from a religious perspective can be seen as a way of fulfilling a duty to help those who are sick or suffering (Eisenhauer & Arslanian-Engoren, 2016; Krupic, 2020; Mostafazadeh-Bora & Zarghami, 2017).

On the other hand, religious beliefs can also influence people’s motivations for nonparticipation (McDonald et al., 2012; Sanderson et al., 2017). For example, while people want biobanks to take account of their religious beliefs, many donors are afraid that some research may conflict with their religious, spiritual, ethical, moral, cultural, or philosophical values (De Vries et al., 2016a; Eisenhauer & Arslanian-Engoren, 2016; Yeary et al., 2020). Such research may include embryonic stem cell research, germ-line gene therapy, human cloning, animal research, creation of immortalized cell lines, or human–animal hybrids that can be seen as ‘playing God’ (Goodson & Vernon, 2004; Heredia et al., 2017; Lewis et al., 2013; Lipworth et al., 2009; Singh et al., 2022). Religion can also oppose the usage of biospecimens for commercial research (Kraft et al., 2018). Another barrier to donation can result from religious beliefs surrounding the ‘sacredness of blood’: that the body should remain whole at burial (Halverson & Ross, 2012; Kowal et al., 2015; Simon et al., 2017; Boise et al., 2017; Aramoana & Koea, 2020). Finally, people’s conviction that it is God who determines who lives or dies from various diseases may hinder donors from participating in clinical trials (Yeary et al., 2020).

Indeed, a literature review conducted by Eisenhauer and Arslanian-Engoren revealed that religious values can influence personal decisions to donate to biobank in various ways. Firstly, while for some individuals biobank research—and genetics in particular—can be perceived as a usurpation of the power of God, they can be afraid that donating violates their belief that the human body is a ‘sacred’ gift from God, while others are concerned that their sample could be used in research that contradicts their values and/or is prohibited by religion. Secondly, individuals who believe that sample donation is a religiously good deed that may help cure illness and reduce suffering are more willing to donate samples for research purposes. Thirdly and finally, while religious beliefs can become a source of moral tension between altruistic motives and religiously doubtful research procedures, they can also influence donors’ preferences for consent type, as religious individuals often opt for study-specific consent (Eisenhauer & Arslanian-Engoren, 2016).

Thus, because biobank research can place donors at risk of ethical, moral, or religious dilemmas and conflicts, it is healthcare professionals (HCPs) who are in a key position to reduce those risks (Persaud & Bonham, 2018). Moreover, since they are often assigned the role of study coordinators, and are responsible for the recruitment of the donors and obtaining consent from participants, they can play a crucial role in supporting biobanking research (Miller & Rosenzweig, 2021; Sanner et al., 2015). On the other hand, the lack of HCPs’ support can hinder the process of collecting and processing biospecimens (Caixeiro et al., 2016).

Biobanking is of special importance in countries like Poland, who have been participating in the activities of the European research infrastructure for biobanking (BBMRI-ERIC) only recently and are trying to establish their own network of biobanks (Witoń et al., 2017). A recent study demonstrated that most Polish biobanks are established in the academic environment, either at universities (42%) or research institutions (30%), while only 10% are run by private companies (Chróścicka et al., 2021). Since the activity of biobanks in Poland is not licensed or registered by the state, and Polish law still lacks both specific legal regulations for biobanking and conducting biomedical research (Krekora-Zając, 2019), the role of HCPs in promoting biobanking research cannot be overestimated.

However, while there is plenty of research on the public’s knowledge and attitudes toward tissue donation for biobank research (for review see: Domaradzki & Pawlikowski, 2019), much less is known about the attitudes of medical students. Meanwhile, future HCPs will be responsible not only for caring for patients, but also for promoting biobanking research and data collection. Simultaneously, while some barriers may hinder HCPs’ support for biobanking, there are no known studies conducted on the association between HCPs’ religious affiliation and their attitudes toward biobanking. While HCPs are well positioned to help build bridges between biobank research and potential donors, their religious beliefs have been recognized as a factor that affects patient treatment (Kørup et al., 2019; Lawrence & Curlin, 2009). Consequently, these beliefs can also influence HCPs’ perception of donation and biobank research. Thus, the current study aimed to assess the association between future HCPs’ religiosity and their attitudes toward biobanking of human biological material for research purposes in Poland.

## Methods

### Study Design

This research included data from an anonymous self-administered online questionnaire regarding medical students’ knowledge and attitudes toward biobanking of human biological material for research purposes.

### Participants and Setting

The study was conducted among medical students during the winter semester at the Poznań University of Medical Sciences (PUMS) in Poland between December 2021 and February 2022. Participants were included if they were enrolled in PUMS and were eager to participate in the study. Invitation to participate in the study was posted on an online platform.

### Research Tools

The questionnaire was elaborated according to the guidelines of the European Statistical System (Eurostat, 2005). First, an online focus group of three research experts (a sociologist, public health specialist, and a biotechnologist) was organized to discuss the list of questions regarding key issues related to biobank tissue donation, drawn from a literature review. Next, a standardized questionnaire was developed and pre-tested via an online platform with ten students with a view to reformulating the four questions, and to have them re-evaluated by two additional external experts: a sociologist and biotechnologist.

The questionnaire was designed to explore issues relating to participation in research biobank: overall attitudes toward research biobanks; willingness to participate in research biobanks, motivation for donation and refusal for research purposes, type of samples participants would be willing to donate for research purposes, and type of research they would be willing to donate to, perceived benefits and risk resulting from biobank research, trust toward biobanking institutions, ethics consent preferences, participant withdrawal, funding, payment for participation.

The questionnaire itself consisted of four sections. The first-asked questions were concerned with students’ knowledge of biobank research. The second section included questions regarding students’ willingness and motivations to donate to a biobank for research purposes. The third section is related to ethical and legal issues related to biobanking of human biological material for research purposes. The last section of the questionnaire included questions concerning students’ demographic characteristics.

Prior to completing the survey, all participants were provided a definition of research biobank (“A biobank is a type of biorepository that collects human biological samples, i.e., bodily fluid, DNA or tissues, and associated information organized in a systematic way to help researchers study how genes, the environment, and lifestyle can affect a person’s health”). This was important since previous research showed that the most members of the general public have either not heard about biobanks (Gaskell et al., 2013; Heredia et al., 2017; Rahm et al., 2013; Tupasela et al., 2010), possess little or no knowledge on medical research involving biobanks (De Vries et al., 2016b; Dive et al., 2020; Domaradzki & Pawlikowski, 2019), or confuse participation in a biobank with medical examinations, i.e., diagnosis or treatment (D’Abramo et al., 2015; Nobile et al., 2013).

After the definition, participants were asked to complete the survey. Since the questionnaire consisted of close-ended questions that asked respondents to choose from a distinct set of pre-defined responses, such as ‘yes/no’ or among set multiple questions where the participants were required to indicate the extent to which they agree or disagree, students were asked to choose a response out of the available options for each question. To facilitate completing the survey, the questionnaire did not include technical jargon and descriptive definitions were used instead. Additionally, most questions contained the possibility to give neutral answer (“I do not know”).

To determine students’ religiosity they were asked the following two questions: ‘What is your present religion, if any?’, and ‘What role does religion play in your life?’. Based on their responses, students were divided into two groups: religious students (students for whom religion was important in their life decisions and choices) and ambivalent/nonreligious (students who either declared separation of their religious beliefs from their life decisions and choices or who felt religion was irrelevant to them).

### Data Collection

The final version of the questionnaire was posted on an online platform and distributed among students of all faculties at PUMS (medicine, medical sciences, pharmacy, and health sciences) via Microsoft Teams: a communication platform used at PUMS for educational purposes during the COVID-19 pandemic. Students received an invitation letter and were informed about the study’s purpose, as well as the voluntary, anonymous, and confidential character of the study. Out of all the 5830 students approached, 1500 (25.72%) completed the questionnaire.

Participants completed self-administered, computer-assisted questionnaires using electronic devices. Questionnaires took approximately 20 min to complete and were collected anonymously.

### Ethical Issues

This study was performed in line with the principles of the Declaration of Helsinki (Sawicka-Gutaj et al., 2022). All students received an invitation letter and informed consent was obtained from all respondents enrolled in the study. Additionally, ethics approval and research governance approval were obtained from the PUMS Bioethics Committee (KB-926/21).

### Data Analysis

The data collected in the questionnaires were verified and checked for completeness, quality, and consistency, and were exported into the statistical package JASP (version 0.16.3.0) and PQStat v.1.8.4. The results are presented as descriptive statistics. Pearson’s Chi-square test and the Fisher’s exact test were used where appropriate to assess differences in the distribution of answers among the groups. Socio-demographic factors that may be associated with students’ declarations concerning the impact of religion on their life decisions and choices were examined through logistic regression analysis. The Mann–Whitney U test was applied to compare the answers to five Likert-scale questions between groups. The effect size was measured with rank-biserial correlation. The 95% confidence interval (95% CI) for rank-biserial correlation was calculated. Median was used as the measure of central tendency for Likert-scale data. Interquartile ranges (IQR) 25th–75th percentile were calculated. A 5% level of significance was used for all the hypothesis tests.

## Results

Table 1 shows detailed characteristics of the participants (*N* = 1500). The sample consisted of 1,190 women (79.3%) and 310 men (20.7%), all of Polish origin. Although students represented a variety of degree courses and years of study, over half were enrolled in their first or second year of study (29% and 22.8%, respectively), and students of the medical faculty (24.1%), physiotherapy (13.8%), and pharmacy (12.4%) predominated. While the majority of respondents declared themselves as Roman Catholics (60.8%), 28% of students declared themselves either as atheists or agnostics. On the other hand, the majority declared that religion was irrelevant for their life (70.6%), while only 30.4% declared that it influences their life decisions and choices.

**Table 1:** Participant demographic characteristics

Characteristics	*N* (%)
*Sex*	
Woman	1190 (79.3)
Man	310 (20.7)
*Year of study*	
1	435 (29)
2	341 (22.8)
3	240 (16)
4	218 (14.5)
5	221 (14.7)
6	45 (3)
*Confession*	
Roman Catholic	912 (60.8)
Other Christian	26 (1.7)
Agnostic	114 (7.6)
Atheist	306 (20.4)
Secular-theist	58 (3.9)
Other	84 (5.6)
*Domicile*	
Up to 10,000 inhabitants	377 (25.1)
10–50,000 inhabitants	244 (16.3)
51–100,000 inhabitants	131 (8.7)
101–500,000 inhabitants	229 (15.3)
Above 500,000 inhabitants	519 (34.6)
*What role does religion play in your life?*	
Significant, it influences my life decisions and choices	133 (8.9)
Rather big, I try to follow religious principle in my life	323 (21.5)
Little, I separate religion from public issues	551 (36.7)
None, it is irrelevant to me	493 (33.9)

While students from all twenty faculties at PUMS were invited to participate in the study, no statistically significant difference between the faculty and students’ religiosity was observed (Table 2).

**Table 2:** Students’ religiosity according to faculty

Faculty	Religious students *N* (%)	Ambivalent/nonreligious students *N* (%)	All students	*p*
Medicine	102 (28.2)	260 (71.8)	362 (100)	ns
Physiotherapy	80 (38.6)	127 (61.4)	207 (100)	
Nursing	55 (38.5)	88 (61.5)	143 (100)	
Pharmacy	53 (28.5)	133 (71.5)	186 (100)	
Electroradiology	17 (27.9)	44 (72.1)	61 (100)	
Medical analytics	20 (28.6)	50 (71.4)	70 (100)	
Dentistry	12 (21.8)	43 (78.2)	55 (100)	
Midwifery	30 (31.6)	65 (68.4)	95 (100)	
Medical rescue	6 (23.1)	20 (76.9)	26 (100)	
Public health	19 (35.8)	34 (64.2)	53 (100)	
Dietetics	14 (28.6)	35 (71.4)	49 (100)	
Medical biotechnology	7 (14.9)	40 (85.1)	47 (100)	
Cosmetology	12 (26.1)	34 (73.9)	46 (100)	
Dental techniques	2 (20)	8 (80)	10 (100)	
Forensic analysis	2 (15.4)	11 (84.6)	13 (100)	
Hearing aid	2 (18.2)	9 (81.8)	11 (100)	
Occupational therapy	5 (35.7)	9 (64.3)	14 (100)	
Optometry	10 (35.7)	18 (78.2)	28 (100)	
Pharmaceutical engineering	8 (33.3)	16 (66.7)	24 (100)	
Total	456 (30.4)	1044 (69.6)	1500 (100)	

*ns* statistically not significant

Table 3 presents the results of logistic regression analysis investigating socio-demographic factors influencing students’ declarations regarding the influence of religion on their life decisions and choices. The variables analyzed include sex, faculty, year of the study, confession (religious affiliation), and domicile size. The findings reveal significant associations between religious affiliation, particularly Roman Catholic and Other Christian denominations, and students’ declarations. In contrast, factors such as sex, faculty, or year of the study did not demonstrate such association. Notably, the impact of domicile size on students’ declarations was observed, primarily among students residing in the smallest towns.

**Table 3:** Logistic regression analysis of the influencing socio-demographic factors on students’ declaration that religion influences their life decisions and choices

Variables	OR	95% CI	*p*
Sex	1.338	0.968–1.851	ns
Faculty	1.011	0.984–1.037	ns
Year of the study	1.046	0.963–1.137	ns
*Confession*			
Secular-theist	1.000		
Agnostic	0.128	0.026–0.636	**< 0.05**
Atheists	0.025	0.003–0.204	**< 0.001**
Roman Catholic	6.243	2.794–13.950	**< 0.00001**
Other Christian	3.900	1.258–12.089	**< 0.05**
Other	1.916	0.731–5.017	ns
*Domicile*			
Above 500,000 inhabitants	1.000		
101–500,000 inhabitants	0.988	0.654–1.491	ns
51–100,000 inhabitants	1.238	0.781–1.963	ns
10–50,000 inhabitants	1.127	0.782–1.623	ns
Up to 10,000 inhabitants	1.594	1.153–2.204	**< 0.01**

The statistically significant results are given in boldface

*ns* statistically not significant

Although most respondents in both groups were familiar with biobanks, religiously indifferent students felt significantly more positive about such institutions, and supported the idea of establishing biobanks in Poland more often (*p* < 0.00001) (Table 4). At the same time, the vast majority of students in both groups were not aware of the existence of research biobank in the country.

**Table 4:** Respondents attitudes toward biobanking

	Religious students	Ambivalent/nonreligious students	*p*
*Have your ever heard about research biobanks?*			ns
Yes	334 (73.2)	774 (74.1)	
No	122 (26.8)	270 (25.9)	
*What are your impressions when you hear a word “biobank”?*			**< 0.00001**
Positive	164 (35.9)	567 (54.3)	
Negative	14 (3.1)	7 (0.7)	
Mixed, both positive and negative	165 (36.2)	195 (18.7)	
I do not know, it is irrelevant to me	113 (27.8)	275 (26.3)	
*Is there any research biobank in Poland?*			ns
Yes	164 (36)	363 (34.8)	
No	2 (0.4)	6 (0.6)	
I don’t know	290 (64.6)	675 (64.6)	
*Should a biobank for research purposes be established in Poland?*			**< 0.00001**
Definitely yes	137 (30.1)	507 (48.6)	
Rather yes	214 (46.9)	433 (41.5)	
Rather no	22 (4.8)	10 (0.9)	
Definitely no	5 (1.1)	0 (0)	
It is irrelevant to me	22 (4.8)	37 (3.5)	
I do not know	56 (12.3)	57 (5.5)	
*What would be your primary motivation for donating your biological material to a research biobank?*			**< 0.00001**
To benefit society and future generations	34 (7.4)	43 (4.1)	
Progress in science, helping in generating new knowledge and development of therapies for various diseases	288 (63.2)	781 (74.8)	
To benefit my family, relatives, and mine own	65 (14.3)	72 (6.9)	
The desire to receive medical treatment/service, i.e., medical check-up or information about the research results	4 (0.9)	5 (0.5)	
The desire to know my health status	49 (10.7)	112 (10.7)	
To establishing good relations with healthcare workers	0 (0)	0 (0)	
The desire to receive financial gratification	4 (0.9)	14 (1.3)	
No answer	12 (2.6)	17 (1.6)	
*What would you expect in exchange of donation samples of your biological material to a research biobank?*			**< 0.05**
Acknowledgments	12 (2.6)	16 (1.5)	
Results	86 (18.9)	163 (15.6)	
Personal health information (detection of disease or genetic predispositions)	318 (69.7)	805 (77.1)	
Financial gratification	18 (4)	35 (3.4)	
Nothing	22 (4.8)	25 (2.4)	

The statistically significant results are given in boldface

*ns* statistically not significant

Religion was also associated with students’ motivations for the hypothetical donation of their biological material. While religious students believed that donation would benefit their families, relatives, and themselves more often (14.3% vs. 6.9%), they were less motivated by the desire to stimulate scientific progress and help in the development of therapies for various diseases (63.2% vs. 74.8%). Some differences were also found in relation to the benefits students expected from donating their biospecimens: while both religious and nonreligious students hoped for ‘personal health information’, the latter chose it more frequently (69.7% vs. 77.1%).

Nonreligious students were more willing to participate in biobanking (Table 5), and were more eager to donate their biospecimens to all types of research mentioned. While the smallest but still statistically significant difference was observed for cancer research, the biggest was found for reproductive cloning. Conversely, religion was not associated with students’ preferences for information, as both groups wished to be informed about the biobanking procedure, including the purpose of the research, duration, and place of sample storage or accessibility, as well as protection of their samples.

**Table 5:** Respondents’ willingness to participate in a research biobanking

	Definitely not	Rather not	I do not know	Rather yes	Definitely yes	Median (IQR)	*p*	Rank-biserial correlation	95% CI for Rank-biserial correlation	
									Lower	Upper
*If you were asked, would you donate the sample of your biological material to a biobank for research purposes?*										
Religious students (RS)	21	58	97	230	50	4 (3–4)	**< 0.001**	0.245	0.184	0.304
Nonreligious students (NRS)	12	55	154	578	245	4 (4–4)				
*To what type of research you would be willing to donate to?*										
Research on the pathogenesis of cancer										
RS	6	6	9	126	309	5 (4–5)	**< 0.001**	0.113	0.050	0.175
NRS	1	7	14	200	822	5 (5–5)				
Research on curable somatic disease										
RS	11	23	18	168	236	5 (4–5)	**< 0.001**	0.166	0.104	0.227
NRS	2	21	29	294	698	5 (4–5)				
Research on incurable genetic diseases										
RS	6	15	14	144	277	5 (4–5)	**< 0.001**	0.138	0.075	0.200
NRS	4	12	22	234	772	5 (4–5)				
Research on psychiatric disorders, i.e., schizophrenia, depression										
RS	12	29	25	158	232	5 (4–5)	**< 0.001**	0.195	0.133	0.256
NRS	6	30	40	244	724	5 (4–5)				
Research on intelligence										
RS	28	94	34	136	164	4 (2–5)	** < 0.001**	0.226	0.164	0.285
NRS	15	97	81	302	549	5 (4–5)				
Research on aggression and violence										
RS	23	75	35	153	170	4 (3–5)	**< 0.001**	0.218	0.157	0.278
NRS	8	86	56	331	563	5 (4–5)				
Research on reproductive cloning										
RS	188	136	44	45	43	2 (1–3)	**< 0.001**	0.304	0.245	0.361
NRS	226	268	119	179	252	3 (2–4)				
Commercial research										
RS	178	142	61	50	25	2 (1–3)	** < 0.001**	0.175	0.112	0.235
NRS	285	315	162	162	120	2 (1–4)				
*What information would you like to receive before donation to a research biobank?*										
Type and the purpose of the research										
RS	0	1	2	23	430	5 (5–5)	ns	0.01	− 0.06	0.07
NRS	1	1	1	48	993	5 (5–5)				
Who and where is conducting the research										
RS	2	15	6	97	336	5 (4–5)	ns	− 0.032	− 0.095	0.032
NRS	3	44	15	245	737	5 (4–5)				
Who is the owner of the biobank										
RS	6	57	24	133	236	4 (4–5)	ns	− 0.38	− 0.101	0.026
NRS	12	143	68	317	504	5 (4–5)				
Duration of storage of my samples										
RS	2	36	10	125	283	5 (4–5)	< 0.1	− 0.047	− 0.110	0.017
NRS	15	93	45	280	611	5 (4–5)				
Where the samples are stored										
RS	3	49	12	144	248	5 (4–5)	< 0.1	− 0.052	− 0.115	0.011
NRS	16	120	60	320	528	5 (4–5)				
Who will have access to samples and research results										
RS	3	10	5	75	363	5 (5–5)	ns	0.012	− 0.052	0.075
NRS	1	26	5	170	842	5 (5–5)				
Conditions for withdrawal samples from the biobank										
RS	2	15	7	81	351	5 (5–5)	ns	0.011	− 0.053	0.074
NRS	3	26	17	185	813	5 (5–5)				
Penalties for researchers who abuse the samples										
RS	1	29	13	99	314	5 (4–5)	ns	0.016	− 0.048	0.079
NRS	7	48	32	224	733	5 (4–5)				
*What are the reasons for your refusal to donate to a research biobank?*										
Physical distance and the necessity to travel										
RS	23	166	34	182	51	4 (2–4)	ns	0.041	− 0.023	0.104
NRS	40	366	61	451	126	4 (2–4)				
The necessity to repeat examination										
RS	15	177	26	189	49	4 (2–4)	**< 0.01**	− 0.083	− 0.146	− 0.020
NRS	50	458	65	387	84	3 (2–4)				
Fear over the safety of the data										
RS	21	134	13	168	120	4 (2–5)	**< 0.001**	− 0.125	− 0.187	− 0.062
NRS	85	382	34	324	219	4 (2–4)				
Fear over unethical use of the sample										
RS	16	71	7	143	219	4 (4–5)	**< 0.001**	− 0.220	− 0.279	− 0.159
NRS	77	294	25	314	334	4 (2–5)				
Fear over the invasive nature of the sampling procedure (pain, sight of blood, needles, and injections)										
RS	128	187	14	83	44	2 (1–4)	**< 0.01**	− 0.096	− 0.158	− 0.032
NRS	374	411	22	152	85	2 (1–2)				
Fear of being infected with HIV										
RS	57	166	17	109	107	3 (2–4)	**< 0.001**	− 0.134	− 0.196	0.071
NRS	219	413	32	170	210	2 (2–4)				
Fear over detection of disease or genetic predispositions										
RS	183	204	16	40	13	2 (1–2)	**< 0.01**	0.091	− 0.153	− 0.027
NRS	499	432	23	71	19	2 (1–2)				
Fear that the data generated from the research can result in stigmatization and discrimination										
RS	80	213	24	85	54	2 (2–4)	**< 0.001**	− 0.096	− 0.161	− 0.036
NRS	260	474	38	181	91	2 (2–4)				
Fear over the commercial use of the samples										
RS	19	95	21	169	152	4 (2.75–5)	**< 0.001**	− 0.131	− 0.192	− 0.068
NRS	59	295	64	368	258	4 (2–4)				
Fear that the government could have the access to the samples										
RS	20	136	26	145	129	4 (2–5)	ns	0.043	− 0.021	0.106
NRS	61	266	52	328	337	4 (2–5)				
Fear that the insurance companies could have the access to the samples										
RS	23	140	40	128	125	4 (2–5)	ns	0.013	− 0.050	0.077
NRS	66	293	79	314	292	4 (2–5)				
Fear that the employees could have the access to the samples										
RS	35	151	38	128	104	4 (2–4)	ns	0.006	− 0.057	0.070
NRS	106	322	62	297	257	4 (2–4)				

The statistically significant results are given in boldface

*ns* statistically not significant

However, religion was also strongly correlated with the reasons future HCPs gave for the refusal to participate in biobanking. Religious students were more concerned over the safety of the data, and the possibility of using their samples either for research that contradict their religious beliefs or commercial research; they were also more fearful of being infected with HIV, and that the data generated from the research would result in stigmatization and discrimination.

Religion was also associated with students’ opinions on the type of tissues they would be willing to donate for research purposes (Table 6). Apart from hair, which was acceptable for both groups, nonreligious students were more willing to donate all other tissues, including blood, saliva, skin, cancer, or reproductive tissues; they were also more eager to donate their organs, after death, including their entire body.

**Table 6:** The impact of religion on the type of tissue being donated for research purposes

	Religious students	Ambivalent/nonreligious students	*p*
*Which of the following tissues would you donate for research purposes?*			
Blood	389 (85.3)	941 (90.1)	**< 0.01**
Salvia	378 (82.9)	918 (87.9)	**< 0.01**
Skin	164 (36)	449 (43)	**< 0.05**
Hair	350 (76.8)	835 (80)	ns
Cancer tissues	275 (60.3)	727 (69.6)	**< 0.001**
Reproductive tissues (sperm, eggs)	78 (17.1)	355 (34)	**< 0.00001**
Embryonic cells left after IVF procedure	43 (9.4)	306 (29.3)	**< 0.00001**
Any type of tissue that is left after the medical procedure	197 (43.2)	648 (62.1)	**< 0.00001**
None of the above	15 (3.3)	6 (0.6)	**< 0.0001**
*Which of the following organs would you donate for research purposes after death?*			
Kidney	206 (45.2)	531 (50.9)	**< 0.05**
Eyes	90 (19.7)	294 (28.2)	**< 0.001**
Brain	105 (23)	376 (36)	**< 0.00001**
Lungs	171 (37.5)	495 (47.4)	**< 0.001**
Liver	196 (43)	521 (49.9)	**< 0.05**
Heart	157 (34.4)	435 (41.7)	**< 0.01**
Bones	102 (22.4)	333 (31.9)	**< 0.001**
Whole body	139 (30.5)	553 (53)	**< 0.00001**
None of the above	133 (29.2)	135 (12.9)	**< 0.00001**

The statistically significant results are given in boldface

*ns* statistically not significant

Religious students had a lower level of trust toward various biobanking institutions, including those run by medical universities, and foreign or private biobanks (Table 7). Moreover, although pharmaceutical companies—particularly foreign ones—were the least trusted institutions in both groups, it was religious students who trusted them even less. Interestingly, although the students who felt strongly attached to their religion were more reluctant to donate to a biobank that would grant access to biospecimens to various institutions, they trusted the government and the police more than nonreligious students. Finally, religious students wanted to be asked for permission at the start of every new research venture more frequently.

**Table 7:** Trust toward biobanking institutions

	Definitely not	Rather not	I do not know	Rather yes	Definitely yes	Median (IQR)	*p*	Rank-biserial correlation	95% CI for Rank-biserial correlation	
									Lower	Upper
*Would you donate the sample of your biological material to research to*										
Medical university										
Religious students (RS)	9	21	17	197	212	4 (4–5)	**< 0.001**	0.165	0.103	0.226
Nonreligious students (NRS)	4	28	20	348	644	5 (4–5)				
Public clinical hospital										
RS	16	68	28	216	128	4 (4–5)	**< 0.001**	0.099	0.036	0.161
NRS	19	126	53	475	371	4 (4–5)				
Private clinical hospital										
RS	23	83	29	220	101	4 (3–4)	**< 0.01**	0.078	0.015	0.141
NRS	35	160	62	503	284	4 (4–5)				
Polish biobank										
RS	17	39	35	265	100	4 (4–4)	**< 0.05**	0.073	0.009	0.136
NRS	25	93	69	555	302	4 (4–5)				
Foreign biobank										
RS	47	118	40	185	66	4 (2–4)	**< 0.001**	0.198	0.137	0.259
NRS	33	176	112	484	239	4 (3–4)				
Private biobank										
RS	50	116	53	183	54	4 (2–4)	**< 0.001**	0.105	0.041	0.167
NRS	67	234	133	433	177	4 (2–4)				
Public biobank										
RS	31	86	54	223	62	4 (2–4)	**< 0.001**	0.136	0.073	0.198
NRS	29	134	125	556	200	4 (3–4)				
Polish pharmaceutical company										
RS	61	122	47	177	49	3 (2–4)	**< 0.01**	0.094	0.030	0.156
NRS	95	226	140	448	135	4 (2–4)				
Foreign pharmaceutical company										
RS	87	143	49	133	44	2 (2–4)	**< 0.001**	0.157	0.095	0.219
NRS	113	264	146	390	131	3 (2–4)				
*Would you donate to a biobank if your biological samples would be accessible to:*										
Only researchers from the institution one donated to										
RS	4	6	10	162	274	5 (4–5)	**< 0.001**	0.137	0.074	0.199
NRS	4	5	10	259	766	5 (4–5)				
Researchers from domestic scientific institutions										
RS	11	34	20	242	149	4 (4–5)	**< 0.001**	0.128	0.065	0.190
NRS	7	73	25	477	462	4 (4–5)				
Researchers from domestic commercial companies, including pharmaceutical industry										
RS	72	161	37	139	47	2 (2–4)	**< 0.01**	0.082	0.018	0.145
NRS	138	311	102	363	130	3 (2–4)				
Researchers from foreign scientific institutions										
RS	38	57	28	233	100	4 (3–4)	**< 0.001**	0.236	0.175	0.295
NRS	28	70	45	491	410	4 (4–5)				
Researchers from foreign commercial companies, including pharmaceutical industry										
RS	100	166	38	117	35	2 (2–4)	**< 0.001**	0.146	0.083	0.207
NRS	159	322	103	332	128	3 (2–4)				
Polish and foreign insurance companies										
RS	207	176	31	30	12	2 (1–2)	ns	0.028	− 0.036	0.091
NRS	467	370	83	81	43	2 (1–2)				
Government institutions										
RS	208	149	42	48	9	2 (1–2)	**< 0.01**	− 0.079	− 0.142	− 0.016
NRS	556	301	78	82	27	1 (1–2)				
Police										
RS	178	133	53	78	14	2 (1–3)	**< 0.01**	− 0.086	− 0.149	− 0.023
NRS	481	301	86	151	25	2 (1–3)				
*When should a research biobank ask the donor for permission to use one’s samples?*										
Before every new research										
RS	13	59	8	113	263	5 (4–5)	**< 0.001**	− 0.169	− 0.230	− 0.106
NRS	51	218	53	266	456	4 (2–5)				
If a new research differs from the original										
RS	10	32	5	118	291	5 (4–5)	**< 0.001**	− 0.108	− 0.171	− 0.045
NRS	31	107	34	308	564	5 (4–5)				
If it is going to be used by researchers outside the institution one donated to										
RS	4	16	7	77	352	5 (5–5)	**< 0.01**	− 0.072	− 0.135	− 0.009
NRS	18	67	16	207	736	5 (4–5)				
If it is going to be used by foreign institutions										
RS	3	19	7	87	340	5 (4–5)	**< 0.001**	− 0.124	− 0.186	− 0.061
NRS	21	103	22	235	663	5 (4–5)				
Does not have to ask if the doner consent while donating										
RS	205	99	25	70	57	2 (1–4)	**< 0.01**	0.098	0.035	0.161
NRS	362	253	110	178	141	2 (1–4)				

The statistically significant results are given in boldface

*ns* statistically not significant

Both groups differed in the preferred type of consent, as religious students opted for study-specific consent more often (57.7% vs. 50.3%) (Table 8). On the other hand, students did not differ in their opinions on the procedures for the protection of the data, withdrawing one’s participation, and financial gratification for donors. Similarly, no differences were found in students’ opinions on who should own donated samples and be entitled to financial benefits steaming from biobank research.

**Table 8:** Ethical and legal issues related to biobanking for research purposes

	Religious students	Ambivalent/nonreligious students	*p*
*What type of consent would you prefer while donating your samples to a research biobank?*			**< 0.01**
Blanket (open-ended permission without any limitations and the need to renewed consent)	56 (12.3)	192 (18.4)	
Specific consent (for one experiment with well-defined aim/before every *research* that involves my samples)	263 (57.7)	525 (50.3)	
Broad consent (general consent for a broad range of future studies but subjected to specified limitations and restrictions stated in the consent form)	55 (12.1)	101 (9.6)	
Tiered consent (individually selected categories of research or research uses, e.g., specific diseases, i.e., cancer or neurological diseases, or research conducted only by specified institutions, i.e., publicly-funded but not private)	34 (7.4)	97 (9.3)	
Consent delegated to bioethical committee	34 (7.4)	102 (9.8)	
I do not know	14 (3.1)	27 (2.6)	
*While donation the samples for research purposes the donors should rather*			< 0.1
Specify the types of research for which their specimens can be used	222 (48.7)	451 (43.2)	
Specify the types of research for which their specimens cannot be used	190 (41.7)	464 (44.4)	
I do not know	44 (9.6)	129 (12.4)	
Samples donated by the donors for research purposes should be			ns
Pseudonymized (reversibly coding, i.e., in case of detecting a disease)	402 (88.2)	951 (91.1)	
Anonymized (irreversibly coding)	36 (7.9)	67 (6.4)	
I do not know	18 (3.9)	26 (2.5)	
*When the donor wants to withdraw from the research their sample should be*			ns
Anonymized (irreversible coded) but available for further research	93 (20.4)	216 (20.7)	
Destroyed	241 (52.9)	556 (53.2)	
It should be prohibited to use those samples in further research	105 (23)	217 (20.8)	
I do not know	17 (3.7)	55 (5.3)	
*Who should possess the property rights to the biosamples donated to a research biobank?*			ns
Biobank	52 (11.4)	135 (12.9)	
Donors	114 (25)	243 (23.3)	
Both biobank and donor	277 (60.7)	612 (58.6)	
I do not know	13 (2.9)	54 (5.2)	
*Who should get the financial benefit stemming from the commercialization of results obtained from the research involving donors’ biosamples donated to a research biobank?*			**< 0.01**
Sponsor of the research/biobank owner	96 (21)	264 (25.3)	
Donors	20 (4.4)	15 (1.4)	
Both biobank and donor	247 (54.2)	543 (52)	
I do not know	93 (20.4)	222 (21.3)	
*Should the donors receive financial gratification for donation their samples?*			ns
Yes	143 (31.3)	321 (30.7)	
No	107 (23.5)	242 (23.2)	
I do not know	206 (45.2)	481 (46.1)	

The statistically significant results are given in boldface

*ns* statistically not significant

## Discussion

Poland is a good example of a country having strong regard for traditional and religious values. Despite declining levels of religious beliefs and practices over the past 20 years, 91% of Poles still declare themselves as believers, with 47% practicing regularly (Centrum Badania Opinii Społecznej, 2020). Additionally, although the number of Poles for whom religion is the main source of moral rules has decreased to 13% (Centrum Badania Opinii Społecznej, 2022), religious faith still influences HCPs’ professional decisions and attitudes toward patients. For example, in 2014, nearly 4000 Polish physicians and medical students signed the *Declaration of faith of Catholic doctors and students of medicine on the sexuality and fertility of human beings,* in which they recognized superiority of ‘God’s law’ over the law of nations (Półtawska, 2015). Consequently, some physicians and nurses object to performing legal abortions, prenatal screening tests, contraceptives, or assisted reproduction techniques (Baranowska et al., 2012; Czekajewska et al., 2022; Zaręba et al., 2020). Alternatively, Pawlikowski et al. (2012) demonstrated that, while religiosity positively affects physicians’ altruism, empathy, and holistic approach toward patients, it often clashes with respect for the patient’s autonomy. Finally, a study on medical students’ motivations for volunteering during the COVID-19 pandemic demonstrated that religious students were more likely to be motivated by altruistic drives than by personal or egoistic motives (Domaradzki & Walkowiak, 2021). Similar results were also found in other countries, where religious beliefs affect HCPs’ attitudes toward euthanasia, physician-assisted suicide, sedation into unconsciousness for terminal patients, withdrawal of life support (Balslev van Randwijk et al., 2020; Wenger & Carmel, 2004), or reproductive health (Awoonor-Williams et al., 2020; Davis et al., 2012).

While some studies did not find a relationship between religious beliefs and donors’ willingness to donate (Merdad et al., 2017; Pawlikowski et al., 2022; Pulley et al., 2008; Singh et al., 2022), this study has identified that religion can play a crucial—albeit indirect—role in shaping future HCPs’ attitudes toward biobanking. Thus, it supports observation made by Machin et al.’ (2020) who stresses the complexity of donation system and situate various body parts and body products within the hierarchy of systems involved in the donation process (tissues and cells, organs, biological systems, the person, family, community, culture, society, and nation) and stress that faith and culture are among the most important dimensions that influence people’s decision to donate.

This research confirms that religion is associated with future HCPs’ decision to participate in biobanking; it demonstrates that nonreligious students felt more positive toward biobanking for research purposes, supported the idea of establishing research biobanks in Poland more often, and were more eager to donate their biosamples for research purposes. However, this should not surprise since research demonstrated that believers are often less positive about technology that nonbelievers (49% and 59%, respectively) (European Commission, 2010). It was also found that religiosity is negatively correlated with science knowledge, interest in science topics and activities, and attitudes toward science (Johnson et al., 2015; McPhetres & Zuckerman, 2018; McPhetres et al., 2021). For example, numerous research demonstrated that greater religiosity is correlated with less favorable views toward such scientific innovations as nanotechnology (Scheufele et al., 2009), stem cell research (Allum et al., 2017), genetic testing (White, 2009; Botoseneanu et al., 2011), gene editing (Critchley et al., 2019), or biobanking (Broekstra et al., 2021).

On the other hand, since the moral teaching of Catholic Church in Poland is focused on ethical issues related to prenatal screening tests, contraceptives, assisted reproduction techniques, abortion, or euthanasia (Chyrowicz, 2021) and there is little, if any, religious teaching activity in the field of biomedical research many people, including ¼ of students enrolled in this study, have never heard about biobanks (Gaskell et al., 2013; Heredia et al., 2017; Krajewska-Kułak et al., 2011; Rahm et al., 2013; Tozzo et al., 2017; Tupasela et al., 2010). Simultaneously, as research biobanks in Poland were established only recently biobanking still rises many ethical, moral, or religious concerns to which religious people can object. Consequently, since religious people are more prone to frame biomedical research as controversial, religion can influence people’s motivations for nonparticipation in biobank research (Broekstra et al., 2021; Goodson & Vernon, 2004; Heredia et al., 2017; Lewis et al., 2013; Lipworth et al., 2009; McDonald et al., 2012; Sanderson et al., 2017; Singh et al., 2022).

This research also shows that religious students were statistically significant less willing to donate most types of tissues (including blood, salvia, skin, or cancer tissues), as well as their organs or entire bodies after death. It can be interpreted by the fact that although both tissue and organ donation and transplantation are permissible within the Catholic faith, in Polish culture, which has been greatly influenced by the Catholicism, the body is often perceived as integral part that should remain whole at burial (Centrum Badania Opinii Społecznej 2019). Moreover, research shows that since some tissues or organs are perceived as sacred or as the very essence of humanity (brain, heart, blood, eyes), some people are afraid that it will not be treated with respect during the research or after an autopsy. For example, since religion affects peoples’ beliefs on brain death (Majchrowicz, 2022; Majchrowicz et al., 2021) it can negatively affect people’s attitude toward brain donation (Aramoana et al., 2020; Halverson & Ross, 2012; Hussen et al., 2017; Kowal et al., 2015; Kraft et al., 2018; Marmamula et al., 2022; Simon et al., 2017). This is in line with previous studies that demonstrated that while people are eager to donate blood, cancerous tissue, skin, bones or/and liver tissue left over after a medical procedure, the most controversial types of biosamples include: brain postmortem, eyes postmortem, human embryos and reproductive tissues (Boise et al., 2017; Goodson & Vernon, 2004; Lewis et al., 2013; Majchrowska et al., 2022; Merdad et al., 2017).

Another important finding is that religious students enrolled in this study were less willing to participate in most types of biobank research, such as into cancer, curable somatic diseases, incurable genetic diseases, or psychiatric disorders. However, this can result from the fact that religious persons are more concerned over research procedures and the purposes for which their biosamples will be used (De Vries et al., 2016a, 2016b; Eisenhauer & Arslanian-Engoren, 2016; Yeary et al., 2020). Research suggests that while most people are willing to donate to research on somatic diseases, including cancer, they are more reluctant to donate to research in genetic disorders or those with a stigmatizing potential, i.e., on mental disorders, but also commercial research, research conducted abroad or research that rise moral concerns, i.e., genetic cloning, gene editing or involving cells from embryos (Barnes et al., 2020; Goodson & Vernon, 2004; Heredia et al., 2017; Lemke et al., 2010; Lewis et al., 2013; Lipworth et al., 2009; Schwartz et al., 2001).

It was also striking to note that religiosity was associated with future HCPs’ motivations for participation: religious students stressed the values of altruism, acting for the greater good, and helping others, stating that biobank research is useful to help the sick or suffering. On the other hand, religiously indifferent students were more orientated toward stimulating scientific progress and the development of therapies. However, this should not surprise, since while most donors’ are primarily motivated by the general feeling of duty and the wish to help others (D’Abramo et al., 2015; Hoeyer, 2010; Lemke et al., 2010; Tozzo et al., 2017) or by the desire to help develop new therapies (Heredia et al., 2017; Lewis et al., 2013; Merdad et al., 2017; Porteri et al., 2014; Rahm et al., 2013), it was shown that religious beliefs in moralizing gods promote various pro-social behaviors (Purzycki et al., 2016). For example, research demonstrated that religion is an important predictor of volunteering and that the imperative of helping others is more common among religious people. It was also shown that more religious people volunteer more frequently and dedicate more hours to voluntary service (Herzog et al., 2020; Niebuur et al., 2018). Moreover, since most religions people perceive donation as an act of charity and love or ‘the gift of life’ religion positively effects blood donation (Beyerlein, 2016; Zangiacomi Martinez et al., 2014).

Similarly, while Khatib et al. (2021) reported that religious views on participation in biobank research were important for 60.5% of Jordanian university students, religion was the main reason for not willing to donate biospecimens for most senior healthcare students in Saudi Arabia (Merdad et al., 2017). Additionally, the views of Malay Muslims in Singapore toward donation and biobanking were negatively shaped by their religious beliefs regarding blood storage (Wong et al., 2004); more than two-thirds of Saudi students declared that religious beliefs influenced their decision toward deceased organ/tissue donation, and that they were among the most important reasons for objecting such donation (Al-Ghanim, 2009). Regarding Turkish students, 16.0% found organ donation religiously inappropriate and many declared that donation did not comply with their religion (Sagiroglu et al., 2015). McDonald et al. (2012) and Sanderson et al. (2017) also reported that respondents with higher levels of religiosity were significantly less likely to provide biospecimens for research. Similar results were found among American breast cancer patients (Sheppard et al., 2018). Finally, in a British study conducted by Lewis et al. (2013), nonbelievers and less religious persons were more interested in donation.

This study also shows that, while religiosity is associated with future HCPs’ perception of risks related to biobanking, it also served as a rationale for nonparticipation. On the other hand, religiously indifferent students were less concerned over various risks resulting from biobank research, including data protection, the possibility of using their samples for research that contradicts their religious beliefs, and the risks of stigmatization and discrimination. However, previous research also showed that although most respondents emphasize benefits resulting from biobank research and believe that it will positively affect entire sociate and future generations, many people are concerned over the risks resulting from participation in a biobank, especially in terms of data protection and using samples against the donors’ will (Gaskell et al., 2013; Igbe & Adebamowo, 2012). Thus, this finding is in line with Hasrizul et al.’s (2017) research: while most religious Malaysians did not perceive donation as immoral, and perceived more benefits and threats resulting from biobanking, still they perceived biobanks as risky. Another study conducted in the same country showed that, although religious Malaysians often perceived biobanking as beneficial, they were also more critical of certain risks underlying its application, often opting for study-specific consent (Amin et al., 2018). Similarly, although religious authorities in Singapore supported participation in biobank research and often defined donation in altruistic terms, they were also reluctant toward the use of health-related data for commercial purposes, and were concerned over the potential for genetic discrimination by private health insurers (Toh et al., 2021).

Similarly, a study conducted in Singapore showed that religious beliefs affected Muslims’ concerns over the selection process, possible ‘discriminating’ potential of genetic testing, and benefit-sharing (Wong et al., 2004). Also religious individuals in Nigeria emphasized the need to ensure that donated samples would not be used for research that contradicts their religious values (Igbe & Adebamowo, 2012). A study conducted in the United States highlighted that some donors were concerned that biobank research conflicting with their religious, cultural, or philosophical beliefs (De Vries et al., 2016a). Although most Jordanians felt positive about donations, over 65% feared their biosamples could be used in religiously prohibited research (Makhlouf et al., 2019). Finally, a recent study conducted among Ghanaians and Nigerians on neurobiobanking demonstrated that over half of respondents did not want to donate their brain because they wanted to ‘go back to God complete’ (Singh et al., 2022). On the other hand, only 12.2% of Saudi participants were concerned that research on human genetics tampered with religion (Al-Jumah et al., 2011).

Another important finding on religiosity is that, while it was negatively associated with medical students’ attitudes toward various biobank research, it was also correlated with their perception of different types of organizations collecting biospecimens for research purposes, as well as preferences for the study-specific consent. However, this should not surprise, since the willingness to donate, preference for broad consent and lower perception of the risks related to the privacy and confidentiality of sample are often positively correlated with trust in research institutions, including biobanks (Domaradzki & Pawlikowski, 2019; Heredia et al., 2017; Sanderson et al., 2017). It was also suggested that scientific institutions, i.e., academic and medical, are trusted more than governmental and commercial biobanks and that national institutions are valued over foreign biobanks (Caulfield et al., 2012; Kaufman et al., 2009; Tupasela et al., 2010). Additionally, a recent study by Pawlikowski et al. (2022) demonstrated that while the willingness to participate in a research biobank was related to general trust in people, it was also related to trust in scientists and doctors. This is in line with our research, as it was religiously indifferent students who were more motivated by the desire to stimulate scientific progress and help in the development of therapies for various diseases and were more willing to participate in biobank research.

Simultaneously, this research confirms that religiosity is often associated with being a biobank nondonor, it also shows that certain religious beliefs can positively influence decisions to participate in biobank research. Indeed, even though religiously indifferent students were more willing to donate, 61.4% of religious students supported the idea of establishing a biobank in Poland, and 54.8% were willing to donate. Additionally, many were motivated by altruistic reasons and believed that it would either help advance scientific progress (63.2%), or benefit their families and relatives (14.3%), or society and future generations (7.4%). What is also important is that the vast majority of religious students supported biobank research concerning pathogenesis of cancer, curable somatic disease, psychiatric disorders, and incurable genetic diseases. Apart from reproductive tissues and embryonic cells left after IVF procedure, they were also willing to donate various type of tissues, including blood (85.3%), salvia (82.9%), hair (76.8%), or cancer tissues (60.3%). Moreover, 43.2% of religious students declared the will to donate any type of tissue left after a medical procedure, and 30.5% declared the will to donate their body after death. Finally, the majority of religious students declared the will to donate those samples to various types of biobanks. Thus, it seems that religion has both positive and negative influence future HCPs’ attitudes toward biobanking: while some religious students did not perceive donation biospecimens for research purposes as immoral and perceived many benefits resulting from biobanking, they still perceived biobanks as risky and ethically problematic.

At the same time, such ambivalent attitudes may result not from religion itself, but from respondents’ uncertainty or misrepresentation of religious regulations on donation, and/or scientific research. Indeed, research shows that while some religious individuals believe that donating one’s blood or organs is an act of mercy and a way of fulfilling the duty to help the sick, others believe that it is not encouraged within their religion (Krupic, 2020; Mostafazadeh-Bora & Zarghami, 2017; Zangiacomi Martinez et al., 2014). For example, while many Nigerians declared that religious acquiescence may be an important motivator to donate for biobank research (Igbe & Adebamowo, 2012), neither Egyptian Muslims nor Christians believed that donating samples for biobanking research goes against their religious beliefs (Abdelhafiz et al., 2019). Also, Egyptian physicians declared that religious beliefs do not constitute a barrier to the establishment and work of biobanks in Egypt if the general values of research ethics are respected (Abdelhafiz et al., 2021). In another study, nearly 75% of Jordanians perceived sample donation as a religiously good deed, and felt more positive about donating their biospecimens for future research (Makhlouf et al., 2019). Finally, Nasrella and Clark (2012) reported that Qatari nationals viewed biobank donation as a charitable act compatible with Islam, and one that helps future generations.

All in all, while this study emphasizes the role of future HCPs in promoting biobanking research and building donors’ trust in biobank research, it also stresses that while setting up a biobank it is important to understand not only the donors’ social, cultural, and religious beliefs, but also how religion affects (future) HCPs’ perception of biobanking. Indeed, while medical students’ religiosity was a significant predictor of their willingness to donate biospecimens and participate in biobanking, it was also associated with their perception of benefits and risks resulting from biobanking, perceived trust toward various biobank institutions, and preferred type of consent.

## Study Limitation

Although to our best knowledge this is one of the few studies on the role of religion in decisions about participating in biobanking of future healthcare professionals, it has a few limitations. Firstly, the sample was rather small, which may have an impact on the generalizability and interpretation of the results. However, because this study was conducted during the second wave of the COVID-19 pandemic, the recruitment process was hindered and reduced the number of respondents. Secondly, because some students lacked interest in the study, and/or were unwilling to discuss their opinions on biobanking and tissue donation, the results represent solely the opinions of those who agreed to participate in the study. Thirdly, since this study covered students from only one Polish medical university, it has a local dimension and the results cannot be generalized to the entire population of future HCPs either in Poznań or in Poland as a whole. Consequently, future studies should compare the findings from a larger group, as well as those from other medical universities in the country. Fourthly, as we did not ask specific questions regarding students’ religious beliefs and attendance, further in-depth studies would be required. Fifthly, while this study focused on future HCPs’ attitudes toward biobanking of human biological material for research purposes it did not asses participants’ knowledge on the topic. However, since over ¼ of all study respondents declared they had never heard about biobanks future studies should also assess medical students’ knowledge on biomedical research and biobanks and compare responses from students who are ‘biobank-aware,’ and ‘biobank-non-aware.’ Finally, because this study analyzed only hypothetical—not actual—decisions to donate, one must remember that actual behaviors and intentions often differ.

However, although limited by scale, scope, and composition of the sample, we believe this is the first study to examine the link between future HCPs’ religiosity and their attitudes toward biobanking; it fills the gap in the research on the topic and may stimulate further studies. By helping to understand how religion is associated with the attitudes of medical students toward biobanking, this study can help identify issues that impede the successful integration of tissue donation and biobank research into routine hospital practices.

## Conclusions

Since religion is an important factor that can affect future HCPs’ perception of donation and scientific research, biobanks should consider the religious beliefs of both the donors and HCPs. Because this research shows that, although in some situations religious beliefs can provide comfort in making the decision to enroll in a biobank, it can also constitute a barrier to biobanking research, it is important to consider religious issues while discussing the development of biobanking ethical guidelines. Moreover, because religious acceptance or permission was shown to be one of the most important factors for promoting public participation in biobanking (Ahram et al., 2014; Amin et al., 2018), and some religious persons point to ministers as persons whom they trust (Halverson & Ross, 2012), a preacher or spiritual care practitioner could be involved in a biobank committee. Additionally, religious leaders could be made available in hospitals and biobanks for those who want to consult their decisions. Finally, in order to overcome the existing knowledge deficits and some negative attitudes toward biobanks, it would be desirable to increase the curriculum on genetics and biotechnology in both secondary and tertiary education.
